# Correction to "Plasma lipid metabolites as biomarkers of early white matter degeneration in Alzheimer's disease"

**DOI:** 10.1002/dad2.70234

**Published:** 2025-12-04

**Shaabanpoor Haghighi A, Nasiri H, Ghayourvahdat A, et al. Plasma lipid metabolites as biomarkers of early white matter degeneration in Alzheimer's disease. *Alzheimers Dement*. 2025;17(4):e70217**. https://doi.org/10.1002/dad2.70217.

In the published article, an error occurred in the Correspondence section regarding the affiliation of Mr. Arman Ghayourvahdat. At the top of the article, his affiliation is correctly listed as:

Inventor Member, Iran Representative Office of the International Federation of Inventors’ Associations (IFIA), Tehran, Iran

However, in the Correspondence section, his affiliation was incorrectly listed as:

Student Research Committee, School of Medicine, Zanjan University of Medical Sciences, Zanjan, Iran

This does not match the correct affiliation and creates a discrepancy within the author information. The affiliation in the Correspondence section should be corrected to match the accurate affiliation already provided in the author list.

We apologize for this error.

